# Law to Prevent Ophthalmia Neonatorum

**Published:** 1898-03

**Authors:** 


### LAW TO PREVENT OPHTHALMIA NEONATORUM.

In an editorial recently the *Journal of the American Medical Association* calls attention to the law which has been passed in several States to compel midwives to report cases of purulent ophthalmia of infancy. The necessity for such a law was first brought forward in New York State by Dr. Lucien Howe of Buffalo, and through his efforts such a law was enacted by the State Assembly. When the American Medical Association met in Milwaukee in 1893, resolutions were adopted by the section of ophthalmology approving of legislation which would compel midwives to promptly report cases of purulent ophthalmia of infancy to some legally qualified practitioner. As a result of this movement there has been an active endeavor by ophthalmologists of all sections to have passed by their States a law looking to this end. As a result of this, laws have been passed in the following States: New York, Maine, Rhode Island, Connecticut, Minnesota, Ohio, Maryland, Missouri, Pennsylvania, Iowa, New Jersey, Illinois, and Michigan.

As many of our readers are not familiar with the wording of the law, we give the one as passed in Ohio, which is about the same as all.

A BILL FOR THE PREVENTION OF BLINDNESS IN THE STATE OF OHIO.

SECTION 1. *Be it enacted by the General Assembly of the State of Ohio*, That should one or both eyes of an infant become inflamed or swollen or show any unnatural discharge at any time within ten (10) days after its birth, it shall be the duty of the midwife, nurse or relative having charge of such an infant to report in writing within six (6) hours to the physician in attendance upon the family, or, in the absence of an attending physician, to the health officer of the city, village or township in which the infant is living at the time, or, in case there is no such officer, to some practitioner of medicine legally qualified to practice in the State of Ohio, the fact that such inflammation, swelling, or unnatural discharge exists.

SEC. 2. Any failure to comply with the provisions of this Act shall be punished by a fine of not less than ten dollars ($10.00) nor more than one hundred dollars ($100.00), or imprisonment for not less than thirty (30) days nor more than six (6) months, or both fine and imprisonment.

The editorial referred to further says: "We call attention to this law for the reason that it should be passed also by the legislatures of other States, and we trust that ophthalmologists who most frequently see these sad results of ignorance and delay of midwives, will take the matter in hand, and also that the medical journals in States without this provision will agitate the subject, so that when their legislatures convene, some move may be made to extend still further the effects of this beneficent law."

We heartily agree with the editorial in commenting upon this subject. We have but to look in our blind asylums to see the ravages of ophthalmia neonatorum to realize the gravity of the disease. It is true that we may not have as many midwives in our State as are found in the North, yet they do exist; therefore, the law is applicable to "those who are present." It is very difficult to obtain any medical laws passed by the Georgia legislature, especially by such a body of men who declare that "any blacksmith can pull teeth," and therefore we fear that it will be some time before medical men can enlighten the understanding of these legislators sufficiently for them to even understand for what the law is intended. The suffering medical profession in Georgia went through a severe struggle in their three years' fight to obtain a recognition between physician and quack. However, we shall not cease our warfare, and will in time urge upon the profession the necessity for the enactment of such a law as will save the eyes of many who now fill our asylums for the blind. R.

